# Impacts of Supply Chain Competition on Firms’ Carbon Emission Reduction and Social Welfare under Cap-and-Trade Regulation

**DOI:** 10.3390/ijerph19063226

**Published:** 2022-03-09

**Authors:** Kelei Xue, Guohua Sun

**Affiliations:** School of Management Science and Engineering, Shandong University of Finance and Economics, Jinan 250014, China; xuekelei@sdufe.edu.cn

**Keywords:** supply chain competition, carbon emission reduction, cap-and-trade, consumer behavior, social welfare

## Abstract

In the carbon neutrality era, firms are facing increasingly intense environmental pressure and market competition. This paper considers two competitive supply chains with consumers’ low-carbon preference under the cap-and-trade regulation, each of which consists of one manufacturer and one retailer. Considering competition or integration in vertical and horizontal directions, four different supply chain structures are modeled. By applying a game-theoretical approach, the equilibrium pricing, carbon emission reduction (CER) level, profit, and social welfare are obtained. Through comparison and analysis, the economic and environmental impacts of supply chain competition are explored. The results show that (1) the carbon quota acts as a kind of financial subsidy and brings direct economic profit to the supply chain, which cannot be used to incentivize the firm to invest in CER technology; (2) the HCVI strategy can bring the highest CER level, the most market demand, and social welfare among the four strategies; (3) for the enterprise and the government, it is recommended to take measures and enact policies to strengthen the vertical integration and horizontal competition between supply chains. Our study can guide firms on how to cope with increasingly fierce industry competition and environmental pressure by adjusting their operational decisions and supply chain structure.

## 1. Introduction

Sustainable development has gradually become the common consensus of the whole society to combat global climate change [1,2,3]. In response to global climate change, 196 parties signed the Paris Agreement at COP 21 in Paris on 12 December 2015, with the goal of net-zero carbon emissions by 2050 [4]. As one of the world’s largest emitters of carbon dioxide, China has pledged to achieve a carbon dioxide emissions peak by 2030 and carbon neutrality before 2060 [5]. The European Union (EU) has also adopted ambitious new targets to reduce greenhouse gas emissions to at least 55% below 1990 levels by 2030 and reach net zero emissions by 2050 [6]. To reduce carbon emissions, China, the EU, the United States, and many other countries have adopted and implemented many types of mechanisms, such as the cap-and-trade regulation [7,8,9]. In a cap-and-trade system, every firm is allocated a carbon emission quota by the government and can sell or buy additional and insufficient carbon credits in the carbon trading market. In 2005, the EU set up the EU Emissions Trading System (EU ETS), which is now the world’s biggest carbon market [10]. In 2021, China also launched its carbon emission trading market in the Shanghai environment and energy exchange [11].

Nowadays, firms face more and more market competition and supply chain competition with the globalization of the economy. With the intensification of environmental pressure and market competition, the enterprise has to invest in carbon emission reduction (CER) technologies to produce environmentally friendly products to appeal to more consumers so as to win more market space. However, the investment in CER technologies can bring a huge financial burden to the firm. The fierce market and supply chain competitions will have important impacts on the firms’ carbon abatement and pricing decisions under cap-and-trade regulation on the one hand. On the other hand, the supply chain competition will also influence the supply chain structure. As an example, H&M has teamed up with its upstream suppliers to compete with other fashion apparel companies, e.g., Uniqlo [12]. Our research motivation also comes from the case of Tesla. A few years ago, the Chinese government introduced Tesla. In October 2019, the first car produced at Tesla’s Shanghai plant rolled off the assembly line. The domestic enterprises of new energy vehicles in China, NIO, BYD, and XPeng all face direct competition from Tesla. The following questions naturally arise: What is the impact of the introduction of Tesla on China’s new energy vehicle industry? For Chinese new energy vehicle firms, how should they adjust their operational strategies and supply chain structure to cope with the competition? For the Chinese government, does the introduction of Tesla contribute to the sustainable development of China’s new energy vehicle industry?

In recent years, an increasing number of consumers show low-carbon preference and are willing to pay more for low-carbon products [13,14,15]. In the first half of 2017, the number of people purchasing green products on JD.com, a well-known e-commerce platform in China, increased by 62.2% year on year. The market average price of environmentally friendly household appliances is 58% higher than the average price of similar products [16]. Therefore, the consumers’ low-carbon preference will have important effects on the firm’s operational decisions and supply chain structures. Motivated by the above cases and facts, it is of great practical and theoretical significance to study the competitive supply chains with consumers’ low-carbon preference under the cap-and-trade regulation. Based on the above analysis, we propose the following research questions:

**Research Question 1:** Considering two competitive supply chains, what are the equilibrium carbon emission reduction and pricing decisions in four different supply chain structures under cap-and-trade regulation?

**Research Question 2:** What are the impacts of supply chain competition (including vertical and horizontal competition) on the manufacturer’s equilibrium CER decision, both firms’ profitability, and social welfare considering the consumers’ low-carbon preference?

**Research Question 3:** Is supply chain competition always bad for the firm? Does there exist the optimal supply chain structure from an economic and social point of view?

To answer the above research questions, in this paper, we explore two competitive supply chains, each of which consists of one manufacturer and one retailer. We assume that the consumer demand is influenced by the product’s price and low-carbon level, as well as the rival product’s low-carbon level. We consider four different supply chain structures (including competition or integration in vertical and horizontal directions). In each of the four supply chain structures, by applying a game-theoretical approach, we obtain the equilibrium pricing, market demand, and CER level of each supply chain. Moreover, we explore the economic and environmental impacts of supply chain competition under cap-and-trade regulation.

The remainder of the paper is organized as follows. In Section 2, the related literature is reviewed. Section 3 describes the problem and model. Four supply chain models and corresponding equilibrium solutions are given in Section 4. Section 5 presents the Model comparison and analysis. Concluding remarks and some directions for future research are provided in Section 6. To make the paper more readable, all proofs are presented in Appendix A.

## 2. Literature Review

This study is closely related to two streams of literature: low-carbon supply chain (LCSC) and supply chain competition. In this section, we will review each stream of literature and highlight the differences between the existing research and our paper.

### 2.1. Low-Carbon Supply Chain

Numerous scholars have studied the topic of the low-carbon supply chain from various perspectives for the past few years. Firstly, some recent papers have analyzed and compared different carbon policies and regulations in an LCSC (e.g., [7,17,18,19,20,21,22,23,24,25]). Benjaafar et al. [20] explored the production issues of a firm under four emission control policies, i.e., carbon offsets, carbon tax, cap-and-trade, and strict carbon caps. He et al. [21] studied the carbon emission and production lot-sizing problem under the carbon tax and cap-and-trade regulations. Li et al. [22] compared absolute-cap and intensity-cap carbon regulations and explored their impacts on the supply chain. Xia et al. [23] investigated carbon emission reduction and pricing policies considering reciprocity preferences under a cap-and-trade system. Chen et al. [24] explored the optimal carbon tax design in an LCSC. Fang et al. [25] examined how carbon tariffs affect the global emission control in a global supply chain. 

Secondly, some studies have researched pricing, carbon emission reduction, and coordination in an LCSC (e.g., [26,27,28,29,30,31]). Bai et al. [28] studied the coordination issue of a low-carbon supply chain with two products under cap-and-trade regulation. Xu et al. [27] revealed that both wholesale price and cost-sharing contracts can coordinate a make-to-order supply chain with green technology. Yang and Chen [29] investigated the impacts of Revenue-Sharing (RS) and Cost-Sharing (CS) schemes under the carbon tax policy and found that RS fails to coordinate the supply chain. Bai et al. [30] explored how the emissions reduction technology investment and risk aversion affect the coordination under the carbon tax policy. Qian et al. [31] developed four contracts and studied a channel coordination issue in a two-echelon sustainable supply chain considering the retailer’s fairness and behavior.

Thirdly, there are also some researchers who have studied the interaction of operations, finance, and environment in an LCSC (e.g., [32,33,34,35,36,37,38,39,40,41,42]). Considering the emission-dependent random demand, Cao and Yu [34] studied the trade credit financing (TCF) and coordination problem in an LCSC under a cap-and-trade mechanism. In another study, Cao et al. [35] compared TCF and bank credit financing (BCF) and found that TCF serves as a unique financing equilibrium. Tang and Yang [36] examined the impacts of BCF and the power structure and found that early payment is a financing equilibrium. Moreover, the government can also provide subsidies and green credit for carbon emission reduction (e.g., [37,38,39,40,41,42]). Li et al. [41] analyzed the impacts of government subsidies based on carbon emission reduction levels. In their study, the government-subsidized low-carbon technology, not low-carbon products. An et al. [42] compared the TCF and green credit financing in a supply chain with carbon emission limits.

### 2.2. Supply Chain Competition

Our paper is most related to the studies that have explored the impacts of supply chain competition on the operational decisions in a sustainable supply chain. Zhu and He [43] studied how the supply chain competition at different levels (the manufacturer level, the retailer level, and the supply chain level) impacts the green product design. Guo et al. [44] examined how retail competition and consumer returns affect green product design in a fashion supply chain. Shen et al. [45] investigated how retail competition and environmental taxes influence the introduction of clean technology in the textiles and apparel supply chains. Liu et al. [46] explored how the retail competition and supply chain competition affect the clean development mechanisms adoption. Considering chain-to-chain competition on both price and carbon emission, Wang et al. [47] studied the design problem of a green supply chain network and proposed a benders decomposition algorithm to handle the large-scale problem. Different from the above studies, our paper not only focuses on the pricing and CER decisions but also investigates the impacts of the supply chain structure on CER, the economy, and the environment.

The competition also influences the design of a sustainable supply chain and its operational efficiency. Li and Li [48] studied the game problem of two supply chains under product sustainability competition and derived the equilibrium structure of the two-chain system. They found that the decentralized supply chain prevails over the centralized supply chain if the supply chain competition is fierce enough. Sim et al. [49] examined the economic and environmental impacts of four market structures (horizontal and/or vertical competition) under the carbon tax policy. They found that market structures that are thought to be more efficient are less efficient from a broader perspective (e.g., considering the environment). Following the above two papers, Deng et al. [50] explored and compared the product sustainability strategies under four similar market structures. They found that the competition intensity between sustainable products has an important impact on the supply chain structure selection. Different from the above papers, our paper investigates the impacts of supply chain competition (competition in horizontal and vertical directions) on CER and social welfare under the cap-and-trade regulation in an LCSC.

### 2.3. Methodology and Contribution

In order to highlight the novelty of our paper more clearly, we provide Table 1 to present the main differences between our work and related studies. Our work is closely related to the research of Sim et al. [49], Deng et al. [50], and Yang et al. [12] Sim et al. [49] constructed a price-sensitive demand function and did not consider the carbon emission reduction competition between two supply chains. Deng et al. [50] developed sustainability sensitive demand function and explored the competition in product sustainability. In our work, we extend the demand function and develop a price and carbon emission-dependent demand function. Moreover, we try to investigate whether the competition at both price and carbon emission levels can change the operational efficiency of different supply chain structures under cap-and-trade regulation. Yang et al. [12] studied the supply chain competition under the cap-and-trade mechanism. However, their research mainly focused on how to design an effective incentive mechanism for the downstream retailer to prevent upstream manufacturer’s horizontal cooperation. Different from Yang et al. [12], our paper considers the horizontal integration and total integration in the LCSC. Moreover, we also explore the environmental impacts of different supply chain structures.

## 3. Model Descriptions

We consider two two-echelon supply chains (labeled as 1 and 2), each of which consists of one manufacturer (he) and one retailer (she). Two manufacturers produce substitutable products under cap-and-trade regulation. Two downstream retailers wholesale products from respective manufacturers and sell them to consumers in the market. As consumers pay more attention to the environment, low-carbon and environmentally friendly products are more and more popular among consumers. For manufacturers, they can invest in CER technology to produce low-carbon products to enhance the competitiveness of their products. The investment cost of CER technology for manufacturers can be expressed by a quadratic function, i.e., $C\left(e_{i}\right)=\frac{1}{2}ke_{i}^{2}$, where $e_{i}$ is manufacturer i’s CER level and k is the cost coefficient. This quadratic cost function is widely used in the relevant literature, e.g., Wei et al. [52], Xue et al. [15] To simplify the analysis and focus on the impacts of supply chain competition on carbon emission reduction, we do not consider the production costs of manufacturers. Symbols and notations used in this paper are concluded in Table 2.

In this paper, we assume that consumers have a low-carbon preference and are more willing to purchase low-carbon products. Two manufacturers invest in CER technology and improve the CER level of their products as a competitive strategy versus their rivals. Thus, the demand function is expressed by

(1) $$q_{i}=a_{i}-bp_{i}+e_{i}-\lambda e_{j},\;i=1,2,\;j=3-i,$$

where b>0 and 0<λ<1. In Equation (1), $a_{i}$ represents the primary market scale of the product i, and b is the sensitivity of the market demand with respect to the price of product i. In order to highlight more the CER competition between two supply chains, this paper will not consider the impact of price competition on the demand. Then, in the demand function, we use parameter λ to measure the competition intensity of CER. A larger value of parameter λ corresponds to intensified carbon emission reduction competition between two supply chains. Following Guo et al. [44], we assume $a_{1}=a_{2}=a>0$ to focus on the case of symmetric competing retailers. This form of demand function is widely used in the relevant literature, such as by Tsay and Agrawal [53] and Yang et al. [12]

Under cap-and-trade regulation, the government gives manufacturers carbon quotas (or cap) G. When manufacturers have a surplus or shortage of carbon credits, they can sell or buy them in the carbon trading market. Therefore, cap-and-trade regulation can be regarded as a kind of financial incentive to promote manufacturers to invest in CER technology. Suppose two manufacturers’ initial carbon emission levels are both e. The carbon emissions amount of manufacturer i is

(2) $$E_{i}=e\left(1-e_{i}\right)q_{i},\;i=1,2.$$

The carbon trading amount of manufacturer i is $T_{i}=E_{i}-G$, where $T_{i}>0$ denotes the manufacturer need to buy carbon credits from the carbon trading market, $T_{i}<0$ denotes the manufacturer can sell carbon credits on the carbon trading market. We use h to represent carbon trading price which is an exogenous variable and determined by the carbon trading market. Therefore, manufacturer i’s profit function is

(3) $$\pi_{mi}\left(w_{i},e_{i}\right)=w_{i}q_{i}-h\left(e\left(1-e_{i}\right)q_{i}-G\right)-C\left(e_{i}\right),\;i=1,2,$$

where the first term is the manufacturer i’s earning from selling low-carbon products to retailer i, the second term is the cost or income from carbon trading, the third term is the investment cost of CER technology of manufacturer i. The profit functions of retailer i and supply chain i are

(4) $$\pi_{ri}\left(p_{i}\right)=\left(p_{i}-w_{i}\right)q_{i},\;i=1,2,$$

(5) $$\pi_{sci}\left(p_{i},e_{i}\right)=p_{i}q_{i}-h\left(e\left(1-e_{i}\right)q_{i}-G\right)-C\left(e_{i}\right),\;i=1,2.$$

We also consider the impact of supply chain competition on social welfare. Then, social welfare is

(6) $$SW=\overset{2}{\underset{i=1}{\sum }}\pi_{sci}\left(p_{i},e_{i}\right)+\overset{2}{\underset{i=1}{\sum }}\int_{0}^{q_{i}}\left(\frac{a+e_{i}-\lambda e_{j}-x}{b}-p_{i}\right)dx,\;i=1,2,\;j=3-i,$$

where the second term denotes the total consumer surplus.

**Figure 1:**
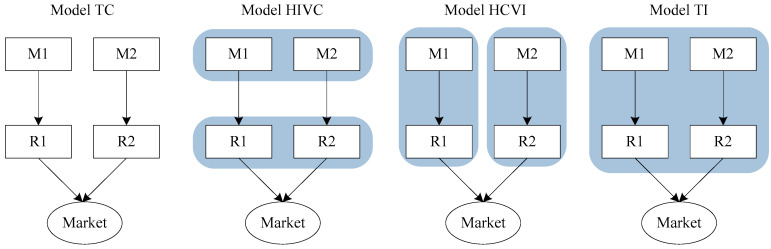
Four supply chain structures (M: Manufacturer, R: Retailer).

In order to explore how the supply chain competition affects the firm’s carbon emission reduction strategy and social welfare under cap-and-trade regulation. We consider four supply chain structures (Figure 1), which are described as follows.

1. **Model TC.** Two supply chains are perfectly competitive, and each supply chain is vertically decentralized. All firms act independently to maximize their respective profit. We describe this scenario as *total competition* (TC);
2. **Model HIVC.** Two supply chains are horizontally integrated and vertically competitive. Two upstream manufacturers and two downstream retailers are integrated as one firm, respectively. This system corresponds to a single decentralized supply chain with one upstream firm and one downstream firm. We describe this scenario as *horizontal integration and vertical competition* (HIVC);
3. **Model HCVI.** Two supply chains are horizontally competitive and vertically integrated. This chain-to-chain competition scenario is equivalent to a duopoly market case. This scenario is described as *horizontal competition and vertical integration* (HIVC);
4. **Model TI.** Two supply chains are totally integrated into both horizontal and vertical directions. All firms in two supply chains act as one single firm and make decisions to maximize their mutual profit. This scenario is described as *total integration* (TI).

## 4. Equilibrium Solutions

In this section, considering the vertical and horizontal competition and integration in two supply chains, the TC, HIVC, HCVI, and TI models are established. The game-theoretical method is used to analyze and solve the above four models. We assume the conditions of $2bk>{\left(1+beh+\lambda \right)}^{2}$ and a−beh>0 hold so that the equilibrium solutions exist in four models. We use the superscript “TC”, “HIVC”, “HCVI”, and “TI” to denote the corresponding variables in four different models. We also use the superscript “*” to mark the optimum value.

### 4.1. Model TC

In model TC, in each supply chain, the manufacturer acts as the Stackelberg game leader and the retailer act as the follower. At the same time, the upstream two manufacturers and the downstream two retailers face respective Nash games. Firstly, the upstream manufacturers decide their wholesale prices and CER levels. Secondly, the downstream retailers decide their retail prices. Thus, the Stackelberg–Nash game problem in model TC can be formulated by

(7) $$\left\{ \begin{array}{c} \{\begin{array}{c} \underset{w_{1},\;e_{1}}{\text{max}}\;\pi_{m1}^{TC}\left(w_{1},e_{1}\right)=w_{1}q_{1}-h\left(e\left(1-e_{1}\right)q_{1}-G\right)-\frac{1}{2}ke_{1}^{2}\\ \underset{w_{2},e_{2}}{\text{max}}\;\pi_{m2}^{TC}\left(w_{2},e_{2}\right)=w_{2}q_{2}-h\left(e\left(1-e_{2}\right)q_{2}-G\right)-\frac{1}{2}ke_{2}^{2} \end{array}\\ p_{1}^{*}\text{ and }p_{2}^{*}\text{ are derived from solving the following problem}\\ \{\begin{array}{c} \underset{p_{1}}{\text{max}}\;\pi_{r1}^{TC}\left(p_{1}\right)=\left(p_{1}-w_{1}\right)q_{1}\\ \underset{p_{2}}{\text{max}}\;\pi_{r2}^{TC}\left(p_{2}\right)=\left(p_{2}-w_{2}\right)q_{2} \end{array} \end{array}\right.$$

where $q_{1}$ and $q_{2}$ are given by Equation (1). Using the backward induction method, the following Lemma 1 can be obtained.

**Lemma** **1.** *In model TC, the optimal equilibrium pricing, market demand, and CER level of each supply chain are given by:*

(8) $$w_{i}^{TC*}=\frac{2k\theta_{1}-eh\theta_{3}\theta_{4}}{2bk+A},$$

(9) $$p_{i}^{TC*}=\frac{k\left(2a+\theta_{1}\right)-eh\theta_{3}\theta_{4}}{2bk+A},\;$$

(10) $$q_{i}^{TC*}=\frac{bk\theta_{2}}{2bk+A},\;$$

*and*

(11) $$e_{i}^{TC*}=\frac{\theta_{2}\theta_{3}}{2bk+A},$$

*where*A=2bk−(1+beh)(1+beh−λ)*,*$\theta_{1}=a+beh$*,*$\theta_{2}=a-beh$*,*$\theta_{3}=1+beh$*,*$\theta_{4}=1+a-\lambda$.

Proofs of Lemma 1 and all other Lemmas, Corollaries, Propositions are provided in Appendix A. Lemma 1 shows that there exists the optimal equilibrium wholesale price, retail price, and CER level for each supply chain. Substituting the optimal solutions given by Lemma 1 into Equations (2), (5), and (6), we can derive the following Lemma 2.

**Lemma** **2.** *In model TC, the following holds:*

*(i)*  *The optimal profit of each supply chain is*
(12) $$\pi_{sci}^{TC*}=Gh+\frac{k\theta_{2}^{2}\left(6bk-\theta_{3}^{2}\right)}{2{\left(2bk+A\right)}^{2}};$$
*(ii)*  *The carbon emissions amount of each supply chain is*
(13) $$E_{i}^{TC*}=\frac{bek\theta_{2}\left(4bk-\theta_{3}\theta_{4}\right)}{{\left(2bk+A\right)}^{2}};$$
*(iii)*  *The social welfare is*
(14) $$SW^{TC*}=2Gh+\frac{\theta_{2}^{2}\left(7bk-\theta_{3}^{2}\right)}{{\left(2bk+A\right)}^{2}}.$$

From Lemmas 1 and 2, we can see that in model TC, all the equilibrium solutions and profits are the same in the two supply chains. This means that when the two supply chains select the same decision-making mode, their optimal decisions will be identical because of symmetric model parameters. Moreover, the carbon quota given by the government has no impact on the optimal equilibrium decisions of the two supply chains system. However, a larger carbon quota can bring more profit for the whole supply chain and more social welfare. Based on the results of Lemmas 1 and 2, we have the following Corollary.

**Corollary** **1.** *In model TC, the following relationships hold:*

*(i)*  $\frac{\partial e_{i}^{TC*}}{\partial \lambda }<0,\;\frac{\partial q_{i}^{TC*}}{\partial \lambda }<0,\;\frac{\partial \pi_{sci}^{TC*}}{\partial \lambda }<0,$$\frac{\partial SW^{TC*}}{\partial \lambda }<0$;
*(ii)*  If $0<k<\frac{eh\left(1+beh\right)}{3}$ *, then* $\frac{\partial w_{i}^{TC*}}{\partial \lambda }>0,\;\frac{\partial p_{i}^{TC*}}{\partial \lambda }>0$*; If* $\frac{eh\left(1+beh\right)}{3}<k<\frac{eh\left(1+beh\right)}{2}$*, then* $\frac{\partial w_{i}^{TC*}}{\partial \lambda }>0,\;\frac{\partial p_{i}^{TC*}}{\partial \lambda }<0$*; If* $k>\frac{eh\left(1+beh\right)}{2}$*, then* $\frac{\partial w_{i}^{TC*}}{\partial \lambda }<0,\;\frac{\partial p_{i}^{TC*}}{\partial \lambda }<0$.

Corollary 1 analyzes how the optimal decisions, profits, and social welfare are affected by the competition intensity of CER. Corollary 1(i) shows that the optimal CER level, market demand, supply chain profit, and total social welfare decrease in the CER competition intensity. A larger λ indicates a greater low-carbon competition between two supply chains. It means that the fiercer of the CER competition, the lower motivation of the manufacturer to invest in CER technology. The less CER technology investment also leads to lower market demand, supply chain profit, and total social welfare.

Corollary 1(ii) shows that if the cost coefficient of CER technology investment (k) is relatively small, the optimal wholesale price and retail price both increase in the CER competition intensity. Because when the CER investment cost is small, the task of emissions reduction becomes easy. Thus, the manufacturer is more willing to invest money to reduce carbon emissions on its own rather than buy extra carbon emissions credits from the carbon trading market. This will lead to the scenario where the products are greener and the manufacturer and retailer both have greater space to increase their wholesale price and retail price even though the competition intensity of CER increases. However, when the CER technology investment is costly, the manufacturer is not willing to invest in CER technology. Consequently, the products are not environmentally friendly. The manufacturer and retailer have to decrease their wholesale price and retail price to attract consumers in order to gain a greater competitive advantage.

### 4.2. Model HIVC

In model HIVC, the integrated upstream manufacturers act as the Stackelberg game leader and decide their wholesale prices and CER levels. The integrated downstream retailers act as the Stackelberg game follower and decide their retail prices. We use the subscript “U” and “D” to denote the upstream manufacturers and downstream retailers, respectively. Thus, the Stackelberg game problem can be formulated by

(15) $$\{\begin{array}{c} \underset{w_{1},w_{2},e_{1},e_{2}}{\max}\;\pi_{U}^{HIVC}\left(w_{1},w_{2},e_{1},e_{2}\right)=\overset{2}{\underset{i=1}{\sum }}\left(w_{i}q_{i}-h\left(e\left(1-e_{i}\right)q_{i}-G\right)-\frac{1}{2}ke_{i}^{2}\right)\\ p_{1}^{*}\;\mathrm{and}\;p_{2}^{*}\;\mathrm{are}\;\mathrm{derived}\;\mathrm{from}\;\mathrm{solving}\;\mathrm{the}\;\mathrm{following}\;\mathrm{problem}\\ \underset{p_{1},p_{2}}{\max}\;\pi_{D}^{HIVC}\left(p_{1},p_{2}\right)=\overset{2}{\underset{i=1}{\sum }}\left(p_{i}-w_{i}\right)q_{i} \end{array},$$

where $q_{i}$ is given by Equation (1). Using the backward induction method, the following Lemma 3 can be obtained.

**Lemma** **3.** *In model HIVC, the optimal equilibrium pricing, market demand, and carbon reduction level of each supply chain are given by:*

(16) $$w_{i}^{HIVC*}=\frac{2k\theta_{1}-eh\left(\theta_{3}-\lambda \right)\theta_{4}}{2bk+B},$$

(17) $$p_{i}^{HIVC*}=\frac{k\left(2a+\theta_{1}\right)-eh\left(\theta_{3}-\lambda \right)\theta_{4}}{2bk+B},\;$$

(18) $$q_{i}^{HIVC*}=\frac{bk\theta_{2}}{2bk+B},$$

*and*

(19) $$e_{i}^{HIVC*}=\frac{\theta_{2}\left(\theta_{3}-\lambda \right)}{2bk+B}.$$

*where* $B=2bk-{\left(1+beh-\lambda \right)}^{2}$.

Lemma 3 shows that there exists the optimal equilibrium wholesale price, retail price, and CER level for each supply chain in model HIVC. Substituting the optimal solutions given by Lemma 3 into Equations (2), (5), and (6), we can derive the following Lemma 4.

**Lemma** **4.** *In model HIVC, the following holds:*

*(i)*  *The optimal profit of each supply chain is*
(20) $$\pi_{sci}^{HIVC*}=Gh+\frac{k\theta_{2}^{2}\left(4bk+B\right)}{2{\left(2bk+B\right)}^{2}};$$
*(ii)*  *The carbon emissions amount of each supply chain is*
(21) $$E_{i}^{HIVC*}=\frac{bek\theta_{2}\left(4bk-\left(1+a+\lambda \right)\theta_{3}+\lambda \theta_{4}\right)}{{\left(2bk+B\right)}^{2}};$$
*(iii)*  *The social welfare is*
(22) $$SW^{HIVC*}=2Gh+\frac{k\theta_{2}^{2}\left(5bk+B\right)}{{\left(2bk+B\right)}^{2}}.$$

From Lemmas 3 and 4, similar to model TC, two supply chains have identical equilibrium solutions. Two supply chains will benefit from the carbon quotas given by the government, which does not affect their optimal decisions. In the following, we explore the impacts of CER competition intensity on the optimal solutions of the supply chain system.

**Corollary** **2.** *In model HIVC, the following relationships hold:*

*(i)*  $\frac{\partial p_{i}^{HIVC*}}{\partial \lambda }<0,$$\frac{\partial e_{i}^{HIVC*}}{\partial \lambda }<0,\;\frac{\partial q_{i}^{HIVC*}}{\partial \lambda }<0,$ $\frac{\partial \pi_{sci}^{HIVC*}}{\partial \lambda }<0,$ $\frac{\partial SW^{HIVC*}}{\partial \lambda }<0;$
*(ii)*  *If*$0<\lambda <\lambda_{1}$*, then*$\frac{\partial w_{i}^{HIVC*}}{\partial \lambda }<0$*; If*$\lambda_{1}<\lambda <1$*, then*$\frac{\partial w_{i}^{HIVC*}}{\partial \lambda }>0$*, where*$\lambda_{1}$*satisfying*$0<\lambda_{1}<1$.

Corollary 2(i) shows that with the increase of the CER competition intensity, the upstream manufacturers have lower motivation to invest in CER technology. Thereby, less CER technology investment leads to lower market demand, supply chain profit, and total social welfare. Different from model TC, the horizontal integration will always lead to the decrease of retail price with the increase of the CER competition intensity. Corollary 2(ii) suggests that the wholesale price first decreases and then increases in CER competition intensity. This conclusion is counter-intuitive. The underlying reason is that when the CER competition intensity is relatively large, both the CER level and retail price decrease, the manufacturer has to increase his wholesale price to maximize profit.

### 4.3. Model HCVI

In model HCVI, two supply chains face the Nash game and each supply chain decides its retail price and CER level. We use the subscript “sci” to denote supply chain i. Thus, the Nash game problem can be formulated by

(23) $$\{\begin{array}{c} \underset{p_{1},e_{1}}{\max}\;\pi_{sc1}^{HCVI}\left(p_{1},e_{1}\right)=p_{1}q_{1}-h\left(e\left(1-e_{1}\right)q_{1}-G\right)-\frac{1}{2}ke_{1}^{2}\\ \underset{p_{2},e_{2}}{\max}\;\pi_{sc2}^{HCVI}\left(p_{2},e_{2}\right)=p_{2}q_{2}-h\left(e\left(1-e_{2}\right)q_{2}-G\right)-\frac{1}{2}ke_{2}^{2} \end{array},$$

where $q_{1}$ and $q_{2}$ are given by Equation (1). Solving the above Nash game problem, the following Lemma 5 can be obtained.

**Lemma** **5.** *In model HCVI, the optimal equilibrium pricing, market demand, and carbon reduction level of each supply chain are given by:*

(24) $$p_{i}^{HCVI*}=\frac{k\theta_{1}-eh\theta_{3}\theta_{4}}{A},$$

(25) $$q_{i}^{HCVI*}=\frac{bk\theta_{2}}{A},\;$$

*and*

(26) $$e_{i}^{HCVI*}=\frac{\theta_{2}\theta_{3}}{A}.$$

Lemma 5 shows that there exist the optimal equilibrium retail price and CER level for each supply chain in model HCVI. Substituting the optimal solutions given by Lemma 5 into Equations (2), (5), and (6), we can derive the following Lemma 6.

**Lemma** **6.** *In model HCVI, the following holds:*

*(i)*  *The optimal profit of each supply chain is*
(27) $$\pi_{sci}^{HCVI*}=Gh+\frac{k\theta_{2}^{2}\left(2bk-\theta_{3}^{2}\right)}{2A^{2}};$$
*(ii)*  *The carbon emissions amount of each supply chain is*
(28) $$E_{sci}^{HCVI*}=\frac{bek\theta_{2}\left(2bk-\theta_{3}\theta_{4}\right)}{A^{2}};$$
*(iii)*  *The social welfare is*
(29) $$SW^{HCVI*}=2Gh+\frac{k\theta_{2}^{2}\left(3bk-\theta_{3}^{2}\right)}{A^{2}}.$$

From Lemmas 5 and 6, two integrated supply chains have identical equilibrium solutions. The two integrated supply chains will benefit from the carbon quota, which does not affect their optimal decisions. In the following, Corollary 3 explores the impacts of CER competition intensity on the optimal solutions of the supply chain system.

**Corollary** **3.** *In model HCVI, the following relationships hold:*$\;\frac{\partial p_{i}^{HCVI*}}{\partial \lambda }<0,$ $\frac{\partial e_{i}^{HCVI*}}{\partial \lambda }<0,\;\frac{\partial q_{i}^{HCVI*}}{\partial \lambda }<0,\;\frac{\partial \pi_{sci}^{HCVI*}}{\partial \lambda }<0,$ $\frac{\partial SW^{HCVI*}}{\partial \lambda }<0$.

Similar to model HIVC, the optimal retail price, market demand, CER level, supply chain profit, and social welfare all decrease in CER competition intensity. Compared with models TC and HIVC, the existence of vertical competition brings more pricing flexibility to the manufacturer and retailer. That is to say, the manufacturer or retailer can increase its price to deal with the intensified CER competition (refer to Corollary 1(ii) and Corollary 2(ii)).

### 4.4. Model TI

In model TI, all firms in two supply chains act as one company and jointly decide the optimal retail prices and CER levels to maximize the whole profit of the supply chain system. Thus, the optimization problem of the supply chain system can be given by

(30) $$\underset{p_{1},p_{2},e_{1},e_{2}}{\max}\pi_{sc}^{TI}\left(p_{1},p_{2},e_{1},e_{2}\right)=\overset{2}{\underset{i=1}{\sum }}\left(p_{i}q_{i}-h\left(e\left(1-e_{i}\right)q_{i}-G\right)-\frac{1}{2}ke_{i}^{2}\right)$$

where $q_{i}$ is given by Equation (1). Solving the above optimization problem, the following Lemma 7 can be obtained.

**Lemma** **7.** *In model TI, the optimal equilibrium pricing, market demand, and carbon reduction level of each supply chain are given by:*

(31) $$p_{i}^{TI*}=\frac{k\theta_{1}-eh\left(\theta_{3}-\lambda \right)\theta_{4}}{B},$$

(32) $$q_{i}^{TI*}=\frac{bk\theta_{2}}{B},$$

*and*

(33) $$e_{i}^{TI*}=\frac{\theta_{2}\left(\theta_{3}-\lambda \right)}{B}.$$

Lemma 7 shows that there exists the optimal and symmetrical retail price and CER level for each supply chain in model TI. Substituting the optimal solutions given by Lemma 7 into Equations (2), (5), and (6), we can derive the following Lemma 8.

**Lemma** **8.** *In model TI, the following holds:*

*(i)*  *The optimal profit of each supply chain is*
(34) $$\pi_{sci}^{TI*}=Gh+\frac{k\theta_{2}^{2}}{2B};$$
*(ii)*  *The carbon emissions amount of each supply chain is*
(35) $$E_{i}^{TI*}=\frac{bek\theta_{2}\left(2bk-\left(\theta_{3}-\lambda \right)\theta_{4}\right)}{B^{2}};$$
*(iii)*  *The social welfare is*
(36) $$SW^{TI*}=2Gh+\frac{k\theta_{2}^{2}\left(bk+B^{2}\right)}{B^{2}}.$$

From Lemmas 7 and 8, the whole integrated supply chain system will benefit from the carbon quota, which does not change its optimal decisions. In the following, we try to explore the impacts of CER competition intensity on the optimal solutions of the supply chain system.

**Corollary** **4.** *In model TI, the following relationships hold:*

*(i)*  $\frac{\partial e_{i}^{TI*}}{\partial \lambda }<0,\;\frac{\partial q_{i}^{TI*}}{\partial \lambda }<0,\;\frac{\partial \pi_{sci}^{TI*}}{\partial \lambda }<0,$$\frac{\partial SW^{TI*}}{\partial \lambda }<0$;

*(ii)*  *If*$0<\lambda <\lambda_{2}$*, then* $\frac{\partial p_{i}^{TI*}}{\partial \lambda }<0$; *If* $\lambda_{2}<\lambda <1$, *then* $\frac{\partial p_{i}^{TI*}}{\partial \lambda }>0$, *where* $\lambda_{2}$*satisfying*$0<\lambda_{2}<1$.

Similar to model TC, the CER level, market demand, supply chain profit, and social welfare decrease in CER competition intensity in model TI. When CER competition intensity is relatively high, the increase of CER level can lead to the increase of retail price.

## 5. Comparisons and Analyses

In this section, we try to compare the optimal decisions, profits, and social welfare in TC and HIVC, HCVI, and TI models to explore the impacts of supply chain competition under cap-and-trade regulation.

### 5.1. Impacts of Horizontal Competition between Two Supply Chains

By comparing the models TC (HCVI) and HIVC (TI), the impacts of horizontal competition on the optimal decisions and profits of the supply chain are derived. Based on the above Lemmas, we have the following Propositions.

**Proposition** **1.** *The optimal CER levels and market demands in different models satisfy:*

*(i)* 
$e_{i}^{TC*}>e_{i}^{HIVC*},\;e_{i}^{HCVI*}>e_{i}^{TI*};$

*(ii)* 
$q_{i}^{TC*}>q_{i}^{HIVC*},\;q_{i}^{HCVI*}>q_{i}^{TI*}.$

From Proposition 1, regardless of the presence of vertical competition, horizontal competition helps to increase the supply chain’s CER level, which also leads to the increase of market demand. This is because the competition between two supply chains urges the manufacturer to increase its product’s CER level to attract consumers in the market. This conclusion is important. The underlying managerial implication is that the government can encourage and facilitate the competition between supply chains to promote carbon emission reduction and sustainable development of the economy.

**Proposition** **2.** *The retail prices in different models satisfy:*

*(i)*  *If* 0<λ<1−beh *, then* $p_{i}^{TC*}>p_{i}^{HIVC*},\;p_{i}^{HCVI*}>p_{i}^{TI*};$

*(ii)*  *If* $1-beh<\lambda \leq 1-\frac{beh}{3}$ *,* $p_{i}^{TC*}>p_{i}^{HIVC*},\;p_{i}^{HCVI*}<p_{i}^{TI*};$

*(iii)*  *If*$1-\frac{beh}{3}<\lambda <1$*,*$p_{i}^{TC*}<p_{i}^{HIVC*},\;p_{i}^{HCVI*}<p_{i}^{TI*}$.

Proposition 2 illustrates that horizontal competition does not always bring a decrease in the retail price. When the CER competition intensity is relatively small, horizontal competition leads to the increase of retail price, regardless of whether the vertical competition exists or not. This is because although the market demand is negatively related to retail price, the supply chain still can increase retail price to maximize its profit. The following proposition summarizes how horizontal competition influences the supply chain profit.

**Proposition** **3.** *The supply chain profits in different models satisfy:*

*(i)* 
$\pi_{sci}^{HCVI*}<\pi_{sci}^{TI*};$

*(ii)*  *When*$-2{\left(beh\right)}^{3}+{\left(beh\right)}^{2}-8bk\left(1-beh\right)>0$*, then*$\pi_{sci}^{TC*}>\pi_{sci}^{HIVC*}$; When$-2{\left(beh\right)}^{3}+{\left(beh\right)}^{2}-8bk\left(1-beh\right)<0$*, then there exits*$\lambda_{3}$*satisfying*$0<\lambda_{3}<1$*, if*$0<\lambda <\lambda_{3}$*,*$\pi_{sci}^{TC*}>\pi_{sci}^{HIVC*}$, if$\lambda_{3}<\lambda <1$*,*$\pi_{sci}^{TC*}<\pi_{sci}^{HIVC*}$.

From Proposition 3, we find that horizontal competition will decrease the supply chain profit without the presence of vertical competition. However, when the CER competition intensity is relatively low, the horizontal competition could increase the supply chain profit with the presence of vertical competition. This conclusion is counter-intuitive. The underlying reason is as follows. The horizontal competition helps increase the CER level. Thus, the manufacturer has to invest more money in CER technology and the supply chain profit may decrease. However, when CER competition intensity is relatively low, the horizontal competition could lead to the increase of the retail price. Hence, the profit from increased revenue could outperform the cost from CER technology investment. Therefore, horizontal competition can help increase the supply chain profit when CER competition intensity is low.

### 5.2. Impacts of Vertical Competition in the Supply Chain

This subsection examines the impacts of vertical competition on equilibrium decisions and profits by comparing the models TC (HIVC) and HCVI (TI). Similarly, Proposition 4 is provided.

**Proposition** **4.** *The optimal CER levels, retail prices, and demands in different models satisfy:*

*(i)* 
$e_{i}^{TC*}<e_{i}^{HCVI*},\;e_{i}^{HIVC*}<e_{i}^{TI*};$

*(ii)* 
$p_{i}^{TC*}>p_{i}^{HCVI*},\;p_{i}^{HIVC*}>p_{i}^{TI*};$

*(iii)* 
$q_{i}^{TC*}<q_{i}^{HCVI*},\;q_{i}^{HIVC*}<q_{i}^{TI*}.$

Proposition 4 reveals that, regardless of whether horizontal competition exists or not, the presence of vertical competition will always decrease the supply chain’s CER level while increasing the retail prices. Consequently, the existence of vertical competition will always decrease the market demand. This conclusion is intuitive. The double marginalization will make the manufacturer and retailer reduce CER technology investment and raise retail prices to maximize their respective profit. From this conclusion, we can derive the following managerial implication. The government should stimulate mutual cooperation in the supply chain to promote carbon emission reduction.

**Proposition** **5.** *The supply chain profits in different models satisfy:*

*(i)* 
$\pi_{sci}^{HIVC*}<\pi_{sci}^{TI*};$

*(ii)*  *When*$-2bk\left(bk-\left(1+beh\right)\right)+\left(bk-beh\right){\left(1+beh\right)}^{2}<0$*, then*$\pi_{sci}^{TC*}<\pi_{sci}^{HCVI*}$; When$-2{\left(beh\right)}^{3}+{\left(beh\right)}^{2}-8bk\left(1-beh\right)>0$*, then there exits*$\lambda_{4}$*satisfying*$0<\lambda_{4}<1$*, if*$0<\lambda <\lambda_{4}$*,*$\pi_{sci}^{TC*}<\pi_{sci}^{HCVI*}$, otherwise,$\pi_{sci}^{TC*}>\pi_{sci}^{HCVI*}$.

Proposition 5 summarizes how vertical competition influences the supply chain profit. From Proposition 5, we find that vertical competition will decrease the supply chain profit without the presence of horizontal competition. However, when the CER competition intensity is relatively high, the vertical competition could increase the supply chain profit with the existence of horizontal competition. This conclusion is interesting and counter-intuitive. The underlying reason is as follows. With the presence of horizontal competition, vertical competition brings the decrease of the CER level and increase of the retail price. When the CER competition intensity is relatively large, the saving from lower CER investment could outperform the reduced revenue because of the increased retail price. Therefore, vertical competition can help increase the supply chain profit when CER competition intensity is large.

### 5.3. Impacts of Competition on the Equilibrium Decisions of Supply Chain

In order to investigate the supply chain competition (coexistence of horizontal and vertical competition) on the equilibrium decisions of the supply chain, we compare models TC, HIVC, HCVI, and TI and derive the following Proposition.

**Proposition** **6.** *The CER levels and market demands in four models satisfy:*

*(i)*  *If*$0<\lambda <\frac{1+beh}{2}$, *then* $e_{i}^{HCVI*}>e_{i}^{TI*}>e_{i}^{TC*}>e_{i}^{HIVC*}$; *If* $\frac{1+beh}{2}<\lambda <1$, $e_{i}^{HCVI*}>e_{i}^{TC*}>e_{i}^{TI*}>e_{i}^{HIVC*}$;
*(ii)* 
$q_{i}^{HCVI*}>q_{i}^{TI*}>q_{i}^{TC*}>q_{i}^{HIVC*}.$

**Figure 2:**
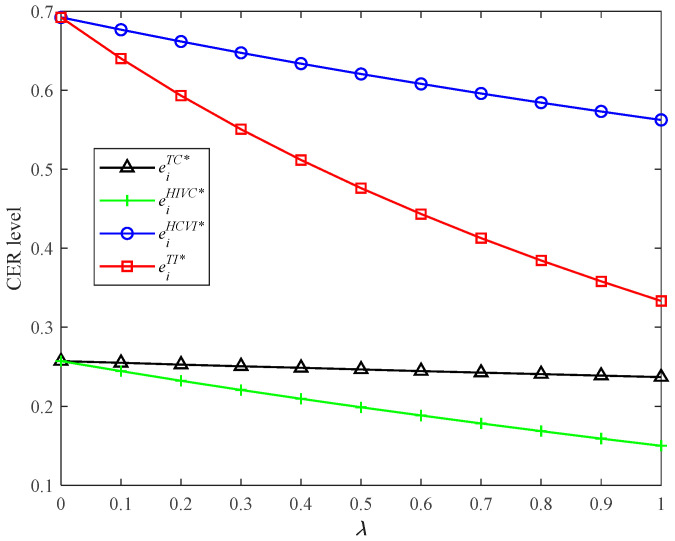
CER levels in four models with different *λ*.

According to Proposition 6, we find that the HCVI model can achieve the highest CER level, while HIVC has the lowest CER level (refer to Proposition 6(i) and Figure 2). This conclusion is interesting and important. This can be explained by two types of effects: horizontal competition effect and vertical competition effect. The first effect is that the horizontal competition will motivate the supply chain to increase the CER level. The second effect is that the vertical competition will prevent the supply chain from increasing the CER level. Thus, the supply chain system with the presence of horizontal competition and vertical integration, i.e., HCVI, has the highest CER level. This implies that it is optimal for the supply chain to choose the HCVI strategy from the perspective of the product’s low-carbon degree.

**Figure 3:**
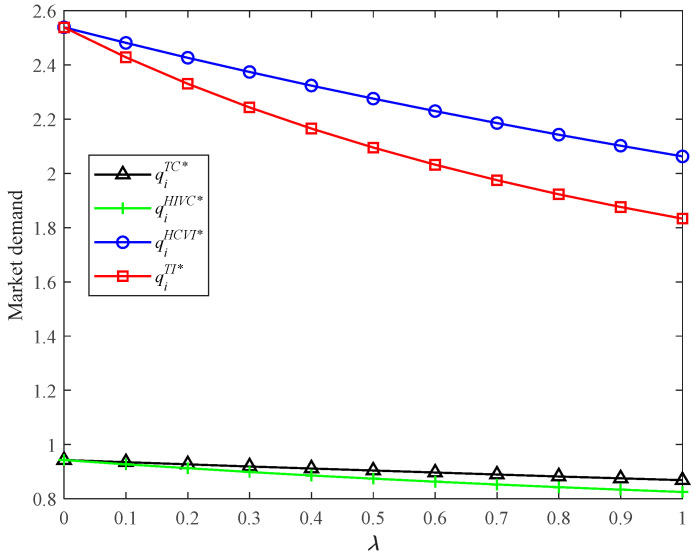
Market demands in four models with different *λ*.

Moreover, the HCVI model can also bring the highest market demand for the supply chain system (refer to Proposition 6(ii) and Figure 3). This can also be explained by the horizontal competition effect and vertical competition effect. Based on the above analysis, we can derive that the presence of horizontal competition and vertical integration brings a higher CER level and market demand for the supply chain system.

**Figure 4:**
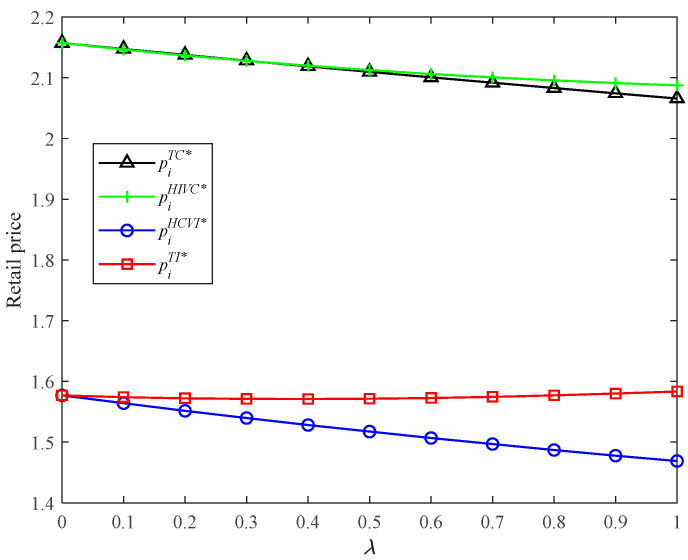
Retail prices in four models with different *λ*.

Now, we turn to analyze the retail prices in four models. Because of complexity, we employ the numerical analysis method. From Figure 4, we find that the HCVI and TI models have lower retail prices than TC and HIVC because of double marginalization. HCVI has the lowest retail price among the four models.

### 5.4. Impacts of Competition on the Economy and Environment

In this subsection, we want to know whether the HCVI and TI models can also bring more profit, social welfare, and less environmental impact. Therefore, we compare profits, total carbon emissions, and social welfare in TC, HIVC, HCVI, and TI models with different CER competition intensities. Because of complexity, the numerical analysis method is used to obtain more managerial implications. We assume that the model parameters satisfy a=5, b=2, e=1, h=1, G=0.7, k=5.5. With the above parameters’ combination, our models are solvable, and our analysis is effective.

#### 5.4.1. Comparison of Supply Chain Profits in Four Models

**Figure 5:**
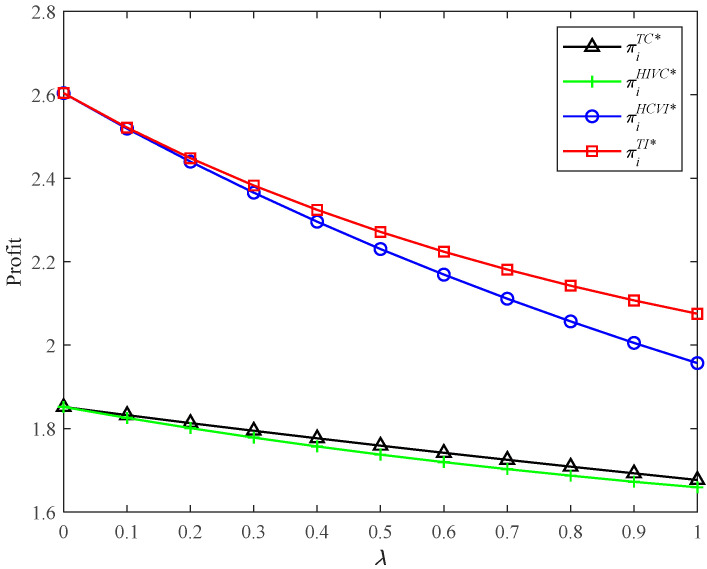
Profits in four models with different *λ*.

Figure 5 intuitively shows that the supply chain profits in HCVI and TI models are more than those in TC and HIVC models, respectively. That is to say, the presence of vertical integration brings more profit for the supply chain system. That is mainly because vertical integration brings higher CER levels, lower retail prices, and higher demand. Hence, a supply chain system with vertical integration achieves more profit. With the presence of vertical competition, the supply chain profit in model TC is bigger than that in model HIVC. The reason is that the increased sales revenue exceeds the increased CER technology investment in model TC. However, without the presence of vertical competition, the supply chain profit in model TI is bigger than that in model HCVI. The reason is that the increased CER technology investment exceeds the increased sales revenue in model HCVI. This shows that the presence of horizontal competition may increase the supply chain profit or decrease the supply chain profit; it depends on the tradeoff between the CER technology investment and the increased income. In conclusion, model TI brings the highest supply chain profit.

#### 5.4.2. Comparison of Total Carbon Emissions in Four Models

**Figure 6:**
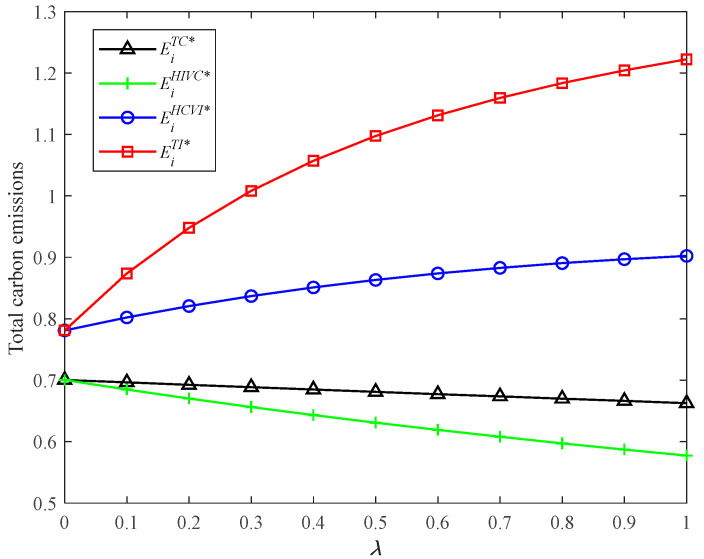
Total carbon emissions in four models with different *λ*.

Figure 6 reveals that the total carbon emissions in HCVI and TI models are more than those in TC and HIVC models, respectively. Combined with the previous analysis, to sum up, the presence of vertical integration not only brings higher CER level and more profit, but also generates more carbon emissions. Similarly, the total carbon emissions in model TC are bigger than those in model HIVC with the presence of vertical competition. However, the total carbon emissions in model TI is bigger than that in model HCVI without the presence of vertical competition. This shows that the presence of horizontal competition may increase or decrease total carbon emissions. It depends on the tradeoff between the increased CER level and the increased production quantity. In conclusion, model TI also brings the most total carbon emissions, in spite of the highest supply chain profit.

#### 5.4.3. Comparison of Social Welfare in Four Models

**Figure 7:**
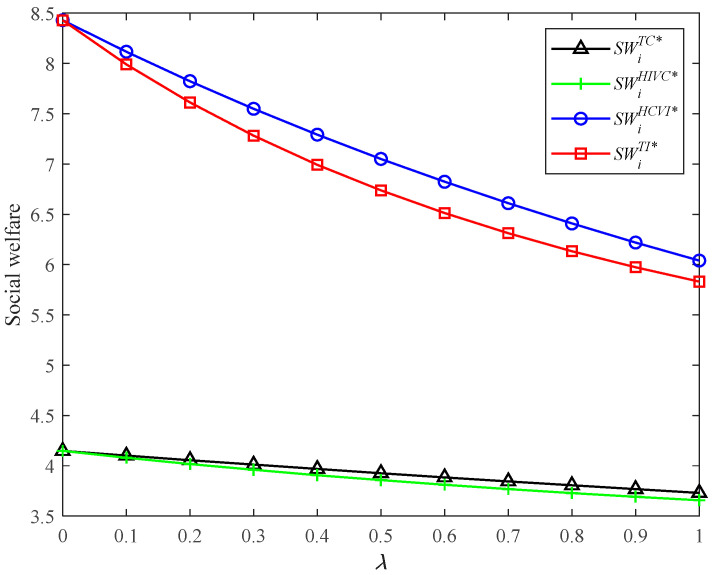
Social welfare in four models with different *λ*.

Figure 7 shows that the social welfare in TI and HCVI is more than that in models TC and HIVC, respectively. The HCVI and TC also bring more social welfare than TI and HIVC, respectively. The presence of vertical integration or horizontal competition brings more social welfare. In particular, the HCVI model brings the most social welfare for the supply chain system. Combining with the previous analysis, the HCVI model can bring the highest CER level, the lowest retail price, and the most market demand. The TI model can bring the most supply chain profit and total carbon emissions. In conclusion, the HCVI model can bring a higher CER level and more social welfare than the TI model, while it brings lower supply chain profit. In other words, the equilibrium supply chain structure, from a comprehensive perspective of economy and environment, does not exist. This finding can provide important managerial implications for firms and governments. For the firm, it is recommended to strengthen vertical integration between upstream and downstream enterprises in the supply chain. For the government, it is suggested to formulate policies to promote competition among supply chains in order to achieve carbon emission reduction and sustainable development.

## 6. Discussions

Through investigating the impacts of supply chain competition, i.e., horizontal competition and vertical competition, on the equilibrium decisions, profits, economy, and environment, we can obtain the following theoretical results and managerial implications which can provide decision-making support for the enterprise and the government.

From the perspective of carbon emissions abatement (environment), HCVI strategy, which is horizontally competitive and vertically integrated can bring the highest CER level, while HIVC strategy (horizontal integration and vertical competition) brings the lowest CER level and social welfare. This finding is different from Sim et al. [49] In their study, the CER levels are the same in different supply chain structures without considering the CER competition. We also find that if the supply chain adopts the TI strategy, the supply chain can obtain the most profits and produce the most carbon emissions among the four strategies. This finding also complements the research of Sim et al. [49].

From the perspective of the economy and society, if the supply chain adopts the HCVI strategy, the supply chain can achieve the most market demand and social welfare among the four strategies. Though the TI strategy can bring the highest profit of the four strategies, it also brings the most negative environmental influence. Among the four strategies, the HIVC strategy brings the worst result to the supply chain. This finding complements the research of Deng et al. [50] In their study, they do not explore the impact of supply chain competition on social welfare. Our finding also complements the study of Li and Li [48], and Yang et al. [12]. They both do not explore the market structures of TI and HIVC, while they have important impacts on the whole supply chain system.

In conclusion, for the enterprise, it is recommended to employ the HCVI strategy to establish its supply chain structure. In other words, the enterprise should strengthen the vertical integration and horizontal competition between supply chains. Taking the case of Tesla as an example, for the Chinese electric vehicle firms confronted with competition from Tesla (or other firms facing a similar situation), on one hand, it is recommended to strengthen the mutual cooperation between upstream and downstream enterprises. On the other hand, electric vehicle firms should also strengthen horizontal competition among enterprises and improve their competitiveness.

For the government, the carbon quota is a kind of financial subsidy and brings direct economic profit to the supply chain, which cannot be used to incentive the firm to invest in CER technology and reduce carbon emissions. This finding complements related research (e.g., [15,28]). The government should also enact policies and take measures to motivate the competition among supply chains. This partly explains why Tesla was introduced in China.

## 7. Conclusions

In this study, we have investigated how the supply chain structure (including horizontal competition and/or vertical competition), affects the firm’s carbon emission reduction strategy and social welfare under cap-and-trade regulation. We consider that consumers have low-carbon preference behavior, and two supply chains compete on the carbon emission reduction level. Depending on whether the supply chain system is integrated vertically and horizontally, four supply chain structures are considered and explored. By applying a game-theoretical approach, we obtain the following core findings and managerial insights:

(1) In four supply chain structures, the more intense the supply chain competition, the lower motivation of the firm to invest in CER technology. The less CER technology investment also leads to lower market demand, supply chain profit, and total social welfare. Under cap-and-trade regulation, the whole supply chain in each of the four market structures benefits from the carbon quota given by the government, which does not affect the equilibrium decisions of the supply chain. A larger carbon quota can bring more gross profit for the whole supply chain and more social welfare;
(2) The presence of horizontal competition or vertical integration will inevitably bring more CER levels and market demand. Under certain conditions, horizontal competition or vertical integration can also generate more profit and social welfare. The underlying managerial insight is that firms should cooperate with their upstream or downstream supply chain partners to reduce carbon emissions while maximizing profits;
(3) When the firm has to face competition from its upstream or downstream partners, horizontal cooperation is never a good choice as it always brings lower CER levels and market demand. In most cases, horizontal cooperation also brings lower profits and social welfare. This finding is important and counter-intuitive. Traditional wisdom is that integration is always of benefit to the supply chain system [48]. However, we find that horizontal competition benefits the supply chain system. When different firms face CER competition, their mutual cooperation will not solve their conflicts, nor will it bring more profits and social welfare;
(4) When the firm has a choice to cooperate with its upstream or downstream partners, vertical integration is always a better choice [54]. Vertical integration always brings a higher CER level, market demand, and more profit and social welfare. The equilibrium supply chain structure, from a comprehensive perspective of the economy and environment, does not exist. The horizontal competition and vertical integration (HCVI) strategy cannot bring the greatest profits but can bring the most environmentally friendly social welfare.

Our paper provides important managerial insights and decision-making references for the firm to establish an appropriate supply chain structure and implement a carbon emission reduction strategy to promote sustainable development. However, there are still some limitations, leaving room for future research. For example, in this study, we only consider that two supply chains compete on CER. In the future, we can consider both price competition and CER competition. For the government, there are also other policies that promote carbon emission reduction, e.g., carbon tax, clean development mechanism [22,46]. Then, we can explore the impacts of supply chain competition on carbon emission reduction strategy under such policies. Furthermore, in the platform economy era, the firm usually has multiple sales channels and faces channel competition. Thus, it will also be interesting to explore the impacts of omni-channel competition on carbon emission reduction strategy in the future.

## Figures and Tables

**Table 1:** Main differences between our works and existing research.

Articles	SCM	SCC	Demand Function	Cap-and-Trade	Focus Point
Bai et al. [30]	M, R	×	PS, SS	×	Profit
Chen et al. [24]	M, R	×	PS	×	Profit
Xue et al. [15]	M, R	×	PCS	×	Profit, Social welfare
Yang et al. [7]	M, R	×	PS	√	Profit
Bai et al. [28]	S, M	×	PCS	√	Profit
Qian et al. [31]	M, R	×	PS, SS	√	Profit
Qi et al. [11]	S, Firm	×	PS, CS	√	Profit
Li et al. [22]	M, R	×	PS, CS	√	Profit
Xu et al. [27]	M, R	×	PS, CS	√	Profit
Xia et al. [23]	M, R	×	PS, CS	√	Profit
Tang and Yang [36]	M, R	×	PS, CS	√	Profit, Social welfare
Guo et al. [44]	M, R	√	SS	×	Profit
Liu et al. [13]	M, R	√	SS	×	Profit
Shen et al. [45]	M, Buyer	√	PS, SS	×	Profit, Social welfare
Sheu and Chen [51]	M, R	√	PS	×	Profit, Social welfare
Zhu and He [43]	M, R	√	PS, SS	×	Profit
Li and Li [48]	S, M	√	SS	×	Profit
Sim et al. [49]	S, M	√	PS	×	Profit, Social welfare
Deng et al. [50]	S, M	√	SS	×	Profit
Yang et al. [12]	M, R	√	PS, CS	√	Profit
Liu et al. [46]	M, R	√	PS	×	Profit
Our paper	M, R	√	PS, CS	√	Profit, Social welfare

SCM: Supply Chain Members; SCC: Supply chain competition; S: Supplier; M: Manufacturer; R: Retailer; PS: Price Sensitive, SS: Sustainability Sensitive; CS: Carbon Sensitive.

**Table 2:** The notations used in this paper.

Notations	Descriptions
$a_{i}$	Initial market demand potential for product $i,\;a_{1}=a_{2}=a$
b	Demand sensitivity coefficient
$q_{i}$	Market demand in supply chain i
λ	Demand sensitivity coefficient to the rival’s carbon emission reduction level
k	Cost coefficient of CER technology investment
$c_{i}$	Production cost of manufacturer $i,\;c_{1}=c_{2}=0$
e	Initial carbon emissions of unit product
$E_{i}$	Carbon emissions of manufacturer i
G	Total carbon quota from the government
$T_{i}$	Carbon trading amount of manufacturer i
$\pi_{ji}^{k}$	the profit of j in supply chain i in model k
$SW^{k}$	Social welfare in model k
Subscript {j, i}	j∈{m, r, sc} denote the manufacturer, retailer, supply chain, respectively; i∈{1, 2} denote the supply chain 1 and 2, respectively
Superscript k	k∈{TC, HIVC, HCVI, TI} denote total competition, horizontal integration and vertical competition, horizontal competition and vertical integration, and total integration, respectively
Decision Variables	
$e_{i}$	Carbon emission reduction level of manufacturer i
$w_{i}$	The wholesale price of product i
$p_{i}$	Retail price of product i

## Data Availability

Not applicable.

## Appendix A

**Proof of Lemmas 1 and** **2.** Firstly, we solve the retailers’ optimization problem. Solving the second derivative of $\pi_{ri}\left(p_{i}\right)$ with respect to $p_{i}$ yields $\frac{\partial^{2}\pi_{ri}^{TC}\left(p_{i}\right)}{\partial {\left(p_{i}\right)}^{2}}=-2b<0$. Hence, $\pi_{ri}\left(p_{i}\right)$ is a concave function with respect to $p_{i}$. By the first order conditions (FOCs), i.e., $\frac{\partial \pi_{r1}^{TC}\left(p_{1}\right)}{\partial p_{1}}=\frac{\partial \pi_{r2}^{TC}\left(p_{2}\right)}{\partial p_{2}}=0$, we derive the optimal response function of the retailer i: $p_{i}^{TC}=\frac{a_{i}+e_{i}-\lambda e_{j}+bw_{i}}{2b}.$ Secondly, we solve the manufacturers’ optimization problem. Substitute $p_{i}^{TC}$ into the manufacturer i’s profit function $\pi_{mi}^{TC}\left(w_{i},e_{i}\right)$. Using $H\left(\pi_{mi}^{TC}\right)$ to denote the hessian matrix of $\pi_{mi}^{TC}\left(w_{i},e_{i}\right)$, we have

$$\frac{\partial^{2}\pi_{mi}^{TC}\left(w_{i},e_{i}\right)}{\partial w_{i}{}^{2}}=-b<0,$$

$$det\left(H\left(\pi_{mi}^{TC}\right)\right)=\left|\begin{array}{cc} \frac{\partial^{2}\pi_{mi}^{TC}\left(w_{i},e_{i}\right)}{\partial w_{i}{}^{2}} & \frac{\partial^{2}\pi_{mi}^{TC}\left(w_{i},e_{i}\right)}{\partial w_{i}\partial e_{i}}\\ \frac{\partial^{2}\pi_{mi}^{TC}\left(w_{i},e_{i}\right)}{\partial e_{i}\partial w_{i}} & \frac{\partial^{2}\pi_{mi}^{TC}\left(w_{i},e_{i}\right)}{\partial e_{i}{}^{2}} \end{array}\right|=bk-\frac{1}{4}{\left(1+beh\right)}^{2}.$$

Based on the condition of $2bk>{\left(1+beh+\lambda \right)}^{2}$, $bk-\frac{1}{4}{\left(1+beh\right)}^{2}>0$ holds. Hence, $H\left(\pi_{mi}^{TC}\right)$ is a negative definite. $\pi_{mi}^{TC}\left(w_{i},e_{i}\right)$ is jointly concave in $w_{i}$ and $e_{i}$. By FOCs, i.e., $\frac{\partial \pi_{m1}^{TC}\left(w_{1},e_{1}\right)}{\partial w_{1}}=\frac{\partial \pi_{m1}^{TC}\left(w_{1},e_{1}\right)}{\partial e_{1}}=\frac{\partial \pi_{m2}^{TC}\left(w_{2},e_{2}\right)}{\partial w_{2}}=\frac{\partial \pi_{m2}^{TC}\left(w_{2},e_{2}\right)}{\partial e_{2}}=0,$ we can derive the optimal wholesale price and CER level of the manufacturer i, which are as follows

$$\{\begin{array}{c} w_{i}^{TC*}=\frac{2k\left(a+beh\right)-eh\left(1+a-\lambda \right)\left(1+beh\right)}{4bk-\left(1+beh\right)\left(1+beh-\lambda \right)}\\ e_{i}^{TC*}=\frac{\left(a-beh\right)\left(1+beh\right)}{4bk-\left(1+beh\right)\left(1+beh-\lambda \right)} \end{array}.$$

Substituting the above optimal decisions into the optimal response price $p_{i}^{TC}$ of the retailer i, we can derive the equilibrium pricing of the retailer i

$$p_{i}^{TC*}=\frac{k\left(3a+ehb\right)-eh\left(1+a-\lambda \right)\left(1+beh\right)}{4bk-\left(1+beh\right)\left(1+beh-\lambda \right)}.$$

Substituting the above optimal decisions of the supply chains into $E_{i}^{TC}$, $\pi_{mi}^{TC}$, $\pi_{ri}^{TC}$, $\pi_{sci}^{TC}$, $SW^{TC}$ and $EcoSW^{TC}$. We can obtain the optimal $E_{i}^{TC*}$, $\pi_{mi}^{TC*}$, $\pi_{ri}^{TC*}$, $\pi_{sci}^{TC*}$, $SW^{TC*}$ and $EcoSW^{TC*}$. □

**Proof of Lemmas 3 and** **4.** Firstly, we solve the downstream retailers’ problem. Using $H\left(\pi_{D}^{HIVC}\right)$ to denote the hessian matrix of $\pi_{D}^{HIVC}\left(p_{1},p_{2}\right)$, we have

$$det\left(H\left(\pi_{D}^{HIVC}\right)\right)=\left|\begin{array}{cc} \frac{\partial^{2}\pi_{D}^{HIVC}\left(p_{1},p_{2}\right)}{\partial p_{1}{}^{2}} & \frac{\partial^{2}\pi_{D}^{HIVC}\left(p_{1},p_{2}\right)}{\partial p_{1}\partial p_{2}}\\ \frac{\partial^{2}\pi_{D}^{HIVC}\left(p_{1},p_{2}\right)}{\partial p_{2}\partial p_{1}} & \frac{\partial^{2}\pi_{D}^{HIVC}\left(p_{1},p_{2}\right)}{\partial p_{2}{}^{2}} \end{array}\right|=4b^{2}>0.$$

Due to $\frac{\partial^{2}\pi_{D}^{HIVC}\left(p_{1},p_{2}\right)}{\partial p_{1}{}^{2}}=-2b<0$, hence $H\left(\pi_{D}^{HIVC}\right)$ is negative definite. $\pi_{D}^{HIVC}\left(p_{1},p_{2}\right)$ is jointly concave in $p_{1}$ and $p_{2}$. By the FOCs, i.e., $\frac{\partial \pi_{D}^{HIVC}\left(p_{1},p_{2}\right)}{\partial p_{1}}=\frac{\partial \pi_{D}^{HIVC}\left(p_{1},p_{2}\right)}{\partial p_{2}}=0$, we can derive $p_{i}^{HIVC}=\frac{a+e_{i}-\lambda e_{j}+bw_{i}}{2b}$. Secondly, we solve the upstream manufacturers’ optimization problem. Substitute $p_{i}^{HIVC}$ into upstream manufacturers’ profit function $\pi_{U}^{HIVC}\left(w_{1},w_{2},e_{1},e_{2}\right)$ and use $H\left(\pi_{U}^{HIVC}\right)$ to denote the hessian matrix of $\pi_{U}^{HIVC}\left(w_{1},w_{2},e_{1},e_{2}\right)$. We can derive the first-order, second-order, third-order leading principal minors and the value of the determinant of $H\left(\pi_{U}^{HIVC}\right)$ are $-b\langle 0,\;b^{2}\rangle 0,\;\frac{1}{4}b\left(1+beh\left(2+beh\right)-4bk+\lambda^{2}\right),\frac{1}{16}\left({\left({\left(1+beh\right)}^{2}-4bk\right)}^{2}-2\left({\left(1+beh\right)}^{2}+4bk\right)\lambda^{2}+\lambda^{4}\right)$, respectively. Due to the condition of $2bk>{\left(1+beh+\lambda \right)}^{2}$, then $\frac{1}{4}b\left(1+beh\left(2+beh\right)-4bk+\lambda^{2}\right)<0$, and $\frac{1}{16}\left({\left({\left(1+beh\right)}^{2}-4bk\right)}^{2}-2\left({\left(1+beh\right)}^{2}+4bk\right)\lambda^{2}+\lambda^{4}\right)>0$ hold. Hence, $H\left(\pi_{U}^{HIVC}\right)$ is negative definite. $\pi_{U}^{HIVC}\left(w_{1},w_{2},e_{1},e_{2}\right)$ is jointly concave in $w_{1}$, $w_{2}$, $e_{1}$ and $e_{2}$. By the FOCs, we can derive the optimal equilibrium wholesale price and CER level which are as follows

$$\{\begin{array}{c} w_{i}^{HIVC*}=\frac{2k\left(a+beh\right)-eh\left(1+a-\lambda \right)\left(1+beh-\lambda \right)}{4bk-{\left(1+beh-\lambda \right)}^{2}}\\ e_{i}^{HIVC*}=\frac{\left(a-beh\right)\left(1+beh-\lambda \right)}{4bk-{\left(1+beh-\lambda \right)}^{2}} \end{array}.$$

Substituting $w_{i}^{HIVC*}$ and $e_{i}^{HIVC*}$ into $p_{i}^{HIVC}$, we have the equilibrium retail price

$$p_{i}^{HIVC*}=\frac{k\left(3a+beh\right)-eh\left(1+a-\lambda \right)\left(1+beh-\lambda \right)}{4bk-{\left(1+beh-\lambda \right)}^{2}}.$$

Substituting $w_{i}^{HIVC*}$, $e_{i}^{HIVC*}$ and $p_{i}^{HIVC*}$ into $q_{i}^{HIVC}$, $E_{i}^{HIVC}$, $\pi_{mi}^{HIVC}$, $\pi_{ri}^{HIVC}$, $\pi_{sci}^{HIVC}$, $SW^{HIVC}$ and $EcoSW^{HIVC}$. We can derive the optimal values of $q_{i}^{HIVC*}$, $E_{i}^{HIVC*}$, $\pi_{mi}^{HIVC*}$, $\pi_{ri}^{HIVC*}$, $\pi_{sci}^{HIVC*}$, $SW^{HIVC*}$ and $EcoSW^{HIVC*}$. □

**Proof of Lemmas 5 and** **6.** Using $H\left(\pi_{sci}^{HCVI}\right)$ to denote the hessian matrix of $\pi_{sci}^{HCVI}\left(p_{i},e_{i}\right)$, we have

$$\frac{\partial^{2}\pi_{sci}^{HCVI}\left(p_{i},e_{i}\right)}{\partial p_{i}{}^{2}}=-2b<0,$$

$$det\left(H\left(\pi_{sci}^{HCVI}\right)\right)=\left|\begin{array}{cc} \frac{\partial^{2}\pi_{sci}^{HCVI}\left(p_{i},e_{i}\right)}{\partial p_{i}{}^{2}} & \frac{\partial^{2}\pi_{sci}^{HCVI}\left(p_{i},e_{i}\right)}{\partial p_{i}\partial e_{i}}\\ \frac{\partial^{2}\pi_{sci}^{HCVI}\left(p_{i},e_{i}\right)}{\partial e_{i}\partial p_{i}} & \frac{\partial^{2}\pi_{sci}^{HCVI}\left(p_{i},e_{i}\right)}{\partial e_{i}{}^{2}} \end{array}\right|=2bk-{\left(1+beh\right)}^{2}.$$

Based on the condition of $2bk>{\left(1+beh+\lambda \right)}^{2}$, $2bk-{\left(1+beh\right)}^{2}>0$ holds. Hence, $H\left(\pi_{sci}^{HCVI}\right)$ is negative definite. $\pi_{sci}^{HCVI}\left(p_{i},e_{i}\right)$ is jointly concave in $p_{i}$ and $e_{i}$. By the FOCs, i.e., $\frac{\partial \pi_{sc1}^{HCVI}\left(p_{1},e_{1}\right)}{\partial p_{1}}=\frac{\partial \pi_{sc1}^{HCVI}\left(p_{1},e_{1}\right)}{\partial e_{1}}=\frac{\partial \pi_{sc2}^{HCVI}\left(p_{2},e_{2}\right)}{\partial p_{2}}=\frac{\partial \pi_{sc2}^{HCVI}\left(p_{2},e_{2}\right)}{\partial e_{2}}0$, we can derive the optimal retail prices and CER levels of two supply chains, which are as follows

$$\{\begin{array}{c} p_{i}^{HCVI*}=\frac{k\left(a+ehb\right)-eh\left(1+a-\lambda \right)\left(1+beh\right)}{2bk-\left(1+beh\right)\left(1+beh-\lambda \right)}\\ e_{i}^{HCVI*}=\frac{\left(a-beh\right)\left(1+beh\right)}{2bk-\left(1+beh\right)\left(1+beh-\lambda \right)} \end{array}.$$

Similarly, substituting $p_{i}^{HCVI*}$ and $e_{i}^{HCVI*}$ into $q_{i}^{HCVI*}$, $E_{i}^{HCVI}$, $\pi_{sci}^{HCVI}$, $SW^{HCVI}$ and $EcoSW^{HCVI}$. We can derive the optimal values of $q_{i}^{HCVI*}$, $E_{i}^{HCVI*}$, $\pi_{sci}^{HCVI*}$, $SW^{HCVI*}$ and $EcoSW^{HCVI*}$. □

**Proof of Lemmas 7 and** **8.** Using $H\left(\pi_{sc}^{TI}\right)$ to denote the hessian matrix of $\pi_{sc}^{TI}\left(p_{1},p_{2},e_{1},e_{2}\right)$, we can derive the first-order, second-order, third-order leading principal minors and the value of the determinant of $H\left(\pi_{sc}^{TI}\right)$ are $-2b<0,\;4b^{2}>0,\;2b\left(1+beh\left(2+beh\right)-2bk+\lambda^{2}\right),{\left({\left(1+beh\right)}^{2}-2bk\right)}^{2}-2\left({\left(1+beh\right)}^{2}+2bk\right)\lambda^{2}+\lambda^{4}$, respectively. Due to the condition of $2bk>{\left(1+beh+\lambda \right)}^{2}$, then $2b\left(1+beh\left(2+beh\right)-2bk+\lambda^{2}\right)<0$ and ${\left({\left(1+beh\right)}^{2}-2bk\right)}^{2}-2\left({\left(1+beh\right)}^{2}+2bk\right)\lambda^{2}+\lambda^{4}>0$ hold. Hence, $H\left(\pi_{sc}^{TI}\right)$ is negative definite. $\pi_{sc}^{TI}\left(p_{1},p_{2},e_{1},e_{2}\right)$ is jointly concave in $p_{1}$, $p_{2}$, $e_{1}$ and $e_{2}$. By the FOCs, we can derive the optimal equilibrium retail prices and CER levels which are follows

$$\{\begin{array}{c} p_{i}^{TI*}=\frac{k\left(a+ehb\right)-eh\left(1+a-\lambda \right)\left(1+beh-\lambda \right)}{2bk-{\left(1+beh-\lambda \right)}^{2}}\\ e_{i}^{TI*}=\frac{\left(a-beh\right)\left(1+beh-\lambda \right)}{2bk-{\left(1+beh-\lambda \right)}^{2}} \end{array}.$$

Substituting $p_{i}^{TI*}$ and $e_{i}^{TI*}$ into $q_{i}^{TI}$, $E_{i}^{TI}$, $\pi_{sc}^{TI}$, $SW^{TI}$ and $EcoSW^{TI}$. We can derive the optimal values of $q_{i}^{TI*}$, $E_{i}^{TI*}$, $\pi_{sc}^{TI*}$, $SW^{TI*}$ and $EcoSW^{TI*}$. □

**Proof** **of** **Corollary** **1.** Due to $\frac{\partial w_{i}^{TC*}}{\partial \lambda }=\frac{\left(a-beh\right)\left(1+beh\right)\left(eh\left(1+beh\right)-2k\right)}{{\left(4bk-\left(1+beh\right)\left(1+beh-\lambda \right)\right)}^{2}}$ and $\frac{\partial p_{i}^{TC*}}{\partial \lambda }=\frac{\left(a-beh\right)\left(1+beh\right)\left(eh\left(1+beh\right)-3k\right)}{{\left(4bk-\left(1+beh\right)\left(1+beh-\lambda \right)\right)}^{2}}$, thus, If $0<k<\frac{eh\left(1+beh\right)}{3}$, then $\frac{\partial w_{i}^{TC*}}{\partial \lambda }>0,\;\frac{\partial p_{i}^{TC*}}{\partial \lambda }>0$; If $\frac{eh\left(1+beh\right)}{3}<k<\frac{eh\left(1+beh\right)}{2}$, then $\frac{\partial w_{i}^{TC*}}{\partial \lambda }>0,\;\frac{\partial p_{i}^{TC*}}{\partial \lambda }<0$; if $k>\frac{eh\left(1+beh\right)}{2}$, then $\frac{\partial w_{i}^{TC*}}{\partial \lambda }<0,\;\frac{\partial p_{i}^{TC*}}{\partial \lambda }<0$. The other relationships are easy to derive, so we omit them here. □

**Proof** **of** **Corollary** **2.** We can easily get $\frac{\partial w_{i}^{HIVC*}}{\partial \lambda }=\left(a-beh\right)\frac{eh{\left(1+beh\right)}^{2}-4k+2\left(2k-eh\left(1+beh\right)\right)\lambda +eh\lambda^{2}}{{\left({\left(1+beh-\lambda \right)}^{2}-4bk\right)}^{2}}$. Assume $f\left(\lambda \right)=eh{\left(1+beh\right)}^{2}-4k+2\left(2k-eh\left(1+beh\right)\right)\lambda +eh\lambda^{2}$, then $\frac{\partial f\left(\lambda \right)}{\partial \lambda }>0$. Thus, f(λ) is increasing in the interval [0,1] and f(0)≤f(λ)≤f(1). Due to f(0)<0 and f(1)>0, then there exist $\lambda_{1}$ satisfying $0<\lambda_{1}<1$, if $0<\lambda <\lambda_{1}$, then $\frac{\partial w_{i}^{HIVC*}}{\partial \lambda }<0$, if $\lambda_{1}<\lambda <1$, then $\frac{\partial w_{i}^{HIVC*}}{\partial \lambda }>0$. The other relationships are easy to derive, so we omit them here. □

**Proof** **of** **Corollary** **3.** The proof of Corollary 3 is easy to derive, so we omit it here. □

**Proof** **of** **Corollary** **4.** The proof of Corollary 4 is similar to the proof of Corollary 2, so we omit it here. □

**Proof** **of** **Proposition** **1.** The proof of the Proposition 1 is easy to derive, so we omit it here. □

**Proof** **of** **Proposition** **2.** It is easy to derive $p_{i}^{TC*}-p_{i}^{HIVC*}=\frac{k\lambda \left(a-beh\right)\left(3\left(1-\lambda \right)-beh\right)}{\left(4bk-\left(1+beh\right)\left(1+beh-\lambda \right)\right)\left(4bk-{\left(1+beh-\lambda \right)}^{2}\right)}$ and $p_{i}^{HCVI*}-p_{i}^{TI*}=\frac{k\lambda \left(a-beh\right)\left(1-\lambda -beh\right)}{\left(2bk-\left(1+beh\right)\left(1+beh-\lambda \right)\right)\left(2bk-{\left(1+beh-\lambda \right)}^{2}\right)}$. Thus, we can prove Proposition 2. □

**Proof** **of** **Proposition** **3.** We can easily get $\pi_{sci}^{TC*}-\pi_{sci}^{HIVC*}=\frac{b{\left(a-beh\right)}^{2}k^{2}\lambda }{{\left(4bk-\left(1+beh\right)\left(1+beh-\lambda \right)\right)}^{2}{\left(4bk-{\left(1+beh-\lambda \right)}^{2}\right)}^{2}}\left(8bk\left(1+beh\right)-2{\left(1+beh\right)}^{3}+3\lambda^{3}-8\left(1+beh\right)\lambda^{2}-\left(16bk-7{\left(1+beh\right)}^{2}\right)\lambda \right)$. Assume $g\left(\lambda \right)=8bk\left(1+beh\right)-2{\left(1+beh\right)}^{3}+3\lambda^{3}-8\left(1+beh\right)\lambda^{2}-\left(16bk-7{\left(1+beh\right)}^{2}\right)\lambda$. Then $\frac{\partial g\left(\lambda \right)}{\partial \lambda }<0$ for λ∈[0,1]. Thus, g(λ) is decreasing in the interval [0,1] and g(1)≤g(λ)≤g(0). Therefore, if g(1)>0, i.e., $-2{\left(beh\right)}^{3}+{\left(beh\right)}^{2}-8bk\left(1-beh\right)>0$, then $\pi_{sci}^{TC*}>\pi_{sci}^{HIVC*}$. If $-2{\left(beh\right)}^{3}+{\left(beh\right)}^{2}-8bk\left(1-beh\right)<0$, then g(1)<0. Due to g(0)>0, then there exist $\lambda_{3}$ satisfying $0<\lambda_{3}<1$, if $0<\lambda <\lambda_{3}$, then $\pi_{sci}^{TC*}>\pi_{sci}^{HIVC*}$, if $\lambda_{3}<\lambda <1$, then $\pi_{sci}^{TC*}<\pi_{sci}^{HIVC*}$. The inequation of $\pi_{sci}^{HIVC*}<\pi_{sci}^{TI*}$ is easy to derive, so we omit it here. □

**Proof** **of** **Proposition** **4.** The proof of the Proposition 4 is easy to derive, so we omit it here. □

**Proof** **of** **Proposition** **5.** The proof of the Proposition 5 is similar to the proof of Proposition 3, so we omit it here. □

**Proof** **of** **Proposition** **6.** Based on Propositions 1, 3, 4, and 5, it is easy to derive, so we omit it here. □
